# The investigators reflect: what we have learned from the Deepening our Understanding of Quality Improvement in Europe (DUQuE) study

**DOI:** 10.1093/intqhc/mzu024

**Published:** 2014-03-18

**Authors:** O. Groene, R. Sunol, on behalf of the DUQuE Project Consortium, N. Klazinga, D.S. Kringos, MJMH Lombarts, T. Plochg, M.A. Lopez, M. Secanell, R. Sunol, P. Vallejo, P. Bartels, S. Kristensen, P. Michel, F. Saillour-Glenisson, F. Vlcek, M. Car, S. Jones, E. Klaus, S. Bottaro, P. Garel, M Saluvan, C. Bruneau, A. Depaigne-Loth, C. Shaw, A. Hammer, O. Ommen, H. Pfaff, O. Groene, D. Botje, C. Wagner, H. Kutaj-Wasikowska, B. Kutryba, A. Escoval, A. Lívio, M. Eiras, M. Franca, I. Leite, F. Almeman, H. Kus, K. Ozturk, R. Mannion, O.A. Arah, M. DerSarkissian, C.A. Thompson, A. Wang, A. Thompson

**Affiliations:** 1 London School of Hygiene and Tropical Medicine, Department of Health Services Research and Policy, 15–17 Tavistock Place, London WC1H 9SH, UK; 2 Avedis Donabedian Research Institute(FAD), Universitat Autonoma de Barcelona, Provenza 293, Barcelona 08037, Spain; 3 Red de Investigaciòn en Servicios de Salud en Enfermedades Crònicas, (REDISSEC), Barcelona, Spain

In 2009, we launched Deepening our Understanding of Quality Improvement in Europe (DUQuE), an ambitious study on the effectiveness of quality improvement systems of hospitals in eight European countries. We believe this to be the most comprehensive and detailed study of quality improvement systems to date in terms of the *a priori* development of a coherent theory to guide the measurement and analytical strategy, the diverse countries involved and the range of standardized data collected (including surveys, chart reviews, administrative data and organizational audit). The papers published in this supplement describe the key findings of the project so far [1]. Here we discuss the overarching lessons emerging from the study.

## Feasibility of data collection in EU healthcare settings

Without the relentless efforts of healthcare professionals and patients to gather data, research on quality improvement would not be possible. Despite the demanding workload of the study, response rates and completion of data collection have been exceptional, reflecting the efforts of country and hospital coordinators. However, there were some lessons about feasibility as the field test was complex and challenging for two reasons. First, in some countries an increasing ‘quality burn-out’ (where professionals are overwhelmed by existing requirements to document and monitor aspects of quality and safety) made it difficult to motivate staff to take on yet another project. Secondly, restrictive research ethics criteria (appropriate for interventional studies, but questionable for observational research) led to substantial delays in advancing the study. These are serious issues of relevance for any future primary data collection in European healthcare settings, in particular when providers are approached by multiple EU projects. The EU itself may be able to take a role by establishing governing mechanisms that increase coordination between research projects addressing similar topics.

## Measuring what is important in quality and safety

Quality and safety evaluations in health care are frequently dominated by what can be easily measured, rather than by what is important. The DUQuE Consortium took a deliberate decision to build a conceptual model and populate it with existing instruments where possible, while developing new ones where needed [2]. These new instruments are crucial outputs of the project and we hope they will undergo further validation. However, quality and safety research needs to go beyond descriptive or prevalence studies. Associational analyses that attempt to quantify the complex relationships between quality systems and healthcare processes and outcomes have the potential to contribute much to our understanding of how quality improvement systems work.

## Quality systems and their impact: which definition of quality?

Quality is typically defined in terms of clinical effectiveness, safety and patient centredness [3]. Accepting this definition raises the question of who in the hospital is in charge of assessing and improving these three domains. In DUQuE we hypothesized that all domains are affected by organizational quality management systems. We were able to demonstrate strong associations between quality management and clinical effectiveness of care, and to a lesser extent for patient safety culture. However, the link with patient reported experience of care remains elusive. It might well be that other factors beyond those conceptualized in our study influence the experience of patients. But just as ensuring clinical effectiveness is an organizational effort (it requires evidence-based procedures, monitoring systems and IT support) the improvement of the patient's experience should be an integral part of quality management systems [4].

## A research agenda for research on quality and safety

A number of important questions emerged from this first set of DUQuE results. First, low baseline performance and high variations on a wide range of quality and safety indicators are a key concern. Further work is needed to understand these variations [5, 6]. Secondly, our research suggests that quality management systems are not always implemented systematically and that the extent to which they support the clinical work may be limited. This, too, is an important finding that merits further investigation. Thirdly, our work demonstrates that a combination of departmental level quality strategies is highly associated with achievement of good practice. This observation may lead to further work on developing criteria to characterize the features of a good quality department, which might be of particular benefit to department leaders and to accreditation agencies. Fourthly, if quality is accepted to embrace dimensions of clinical effectiveness, safety and patient-centredness, then further work should address how best to improve patient's experience with care. Fifthly, our research on patient involvement in quality management is sobering. Levels of patient involvement are low and seem tokenistic. How patients can be substantively involved and how to reap the benefits of their involvement urgently requires further work.

## Policy implications

From a policy perspective, DUQuE raises several questions of which a few are mentioned here. In the context of the EU Directive on the rights of patients to cross-border care, policy-makers have wondered whether patient care is more variable in some countries than in others [7]. Decomposing the sources of variation is a challenging task but our results so far suggest a wider variation within countries than between them. These results have implications about the information that should be available to patients choosing a care provider in Europe. To assist purchasing agencies and hospital managers we summarized the key lessons of the project in an ‘An evidence-based guide on effective quality and safety strategies’ [8]. We are well aware that evidence alone doesn't lead to decisions, implementation or improvement. However, we hope that this guide and the research evidence it is based upon will provide the opportunity for a fresh reassessment of what works, what might be modified and where further research is needed.

## Funding

The study, ‘Deepening our Understanding of Quality Improvement in Europe (DUQuE)’ has received funding from the European Community's Seventh Framework Programme (FP7/2007–2013) under grant agreement no. 241822. Funding to pay the Open Access publication charges for this article was provided by European Community's Seventh Framework Programme (FP7/2007–2013) under grant agreement no. 241822.
